# Clinical, Sleep, and Chronobiological Characteristics of Children with Smith–Magenis Syndrome Under Treatment for Sleep Disorders

**DOI:** 10.3390/children12111471

**Published:** 2025-10-31

**Authors:** Marion Comajuan, Aurore Guyon, Véronique Raverot, Marie-Noelle Babinet, Julien Lioret, Lisa Brunel, Bruno Claustrat, Caroline Demily, Patricia Franco

**Affiliations:** 1 Service d’Epileptologie Clinique, des Troubles du Sommeil et de Neurologie Fonctionnelle de L’enfant, Hospices Civiles de Lyon, 69677 Bron, France; 2 Centre de Recherche en Neurosciences de Lyon, Physiologie Intégrative des Systèmes D’éveil du Cerveau, Equipe Waking, Inserm UMRS 1028, CNRS UMR 5292, Université Claude Bernard Lyon 1, Université de Lyon, 69675 Lyon, France; 3 Centre de Biologie Est, Service de Biochimie et Biologie Moléculaire, Hospices Civils de Lyon, 69677 Bron, France; 4 GénoPsy, Centre de Référence Maladies Rares Troubles du Comportement d’Origine Génétique (GénoPsy-Lyon), LeVinatier Psychiatrie Universitaire Lyon Métropole et UMR 5229, CRNS & Université Lyon 1, 95 Boulevard Pinel, 69678 Bron, France; 5 Laboratoire d’Hormonologie, Inserm U846, Centre de Médecine Nucléaire, Hospices Civils de Lyon, 69677 Bron, France

**Keywords:** sleep, pediatric, Smith–Magenis syndrome, melatonin, polysomnography, actigraphy, chronobiology, behavior problems

## Abstract

**Highlights:**

**What are the main findings?**

- Children with Smith–Magenis syndrome showed persistent short and poor-quality sleep despite treatment.
- Very high melatonin levels persisted after treatment withdrawal, suggesting possible iatrogenic accumulation in children receiving very high doses, whose melatonin metabolism (potentially involving slow metabolizers) remains poorly understood.

**What are the implications of the main findings?**

- Routine monitoring of salivary melatonin and personalized dose titration could optimize treatment, avoid overexposure, and improve both sleep and behavior.
- Non-pharmacological approaches such as controlled light therapy and other chronobiotic strategies may represent promising complementary or alternative options to improve circadian alignment in SMS.

**Abstract:**

**Background/Objectives:** Smith–Magenis Syndrome (SMS) is a rare neurodevelopmental disorder characterized by severe sleep disturbances and an advanced melatonin rhythm. Current treatments, mainly exogenous melatonin and β-blockers, have not been evaluated in children. This study aimed to characterize the clinical, sleep, and chronobiological profiles of children with SMS under treatment and to assess the effects of melatonin and β-blockers. **Methods:** In this prospective, single-center study, 20 children with genetically confirmed SMS (aged 5–13 years; 55% female) underwent 15-day home actimetry and 48 h hospitalization, during which questionnaires, polysomnography (PSG), and salivary melatonin/cortisol profiling were performed. Melatonin and psychostimulants were discontinued 36 h before hospitalization. **Results:** Overall, 95% of children received melatonin and 20% β-blockers. Despite treatment, insomnia was reported in 90%, excessive daytime sleepiness in 65%, and learning problems in 90%. On actimetry, melatonin improved the mean nocturnal awakening duration (1.4 vs. 2.3 min, *p* = 0.040), wake-up time (06:50 vs. 06:11, *p* = 0.004), and longest continuous sleep episode (398 vs. 317 min, *p* = 0.040), but had little effect on the total sleep time, efficiency, and midpoint of sleep. Very high daytime salivary melatonin persisted (median peak 169.50 pg/mL) despite the last exogenous melatonin intake being more than 48 h prior to sampling, suggesting possible iatrogenic accumulation. The median peak in melatonin occurred at 11:57 and that in cortisol at 08:22. In children with β-blockers, there was a tendency toward an earlier melatonin peak but also toward delayed sleep onset, increased nocturnal awakenings, and reduced total sleep. **Conclusions:** Children with SMS showed persistent sleep difficulties and an advanced circadian phase despite sleep treatments. Exogenous melatonin provides partial benefit but may lead to daytime accumulation, while β-blockers may have adverse sleep effects despite beneficial effects on melatonin peak secretion, warranting further study.

## 1. Introduction

Smith–Magenis Syndrome (SMS) (OMIM#182290) is a complex disorder characterized by severe neuropsychobehavioral problems, including language delays, intellectual developmental disorders, behavioral problems, and sleep disorders [1,2].

It is a rare disease with a prevalence of 1/15,000 to 1/25,000 [3,4]. In 90% of cases, this syndrome is associated with a microdeletion on the short arm of chromosome 17. More rarely, it involves a mutation of the *RAI1* gene, located in the same chromosomal region [5,6]. *RAI1* is a dosage-sensitive gene expressed in many tissues and acting as a transcriptional regulator, with involvement in neurodevelopment, behavioral function, and circadian rhythms [7].

Sleep disorders observed in SMS patients are characterized by early sleep onset and awakenings during the night, suggesting a phase advance [8]. These disorders could be the consequence of more general dysregulation of the circadian system, inducing a shift in the melatonin secretion profile [9,10]. In healthy individuals, this hormone presents an endogenous circadian rhythm that is synchronized to the light–dark cycle [11]. Serum melatonin concentrations usually peak between 2 and 4 a.m. and vary with age. These levels, which are highest in children aged 1–3 years (and in girls), gradually decline with age [12]. The rise in the evening facilitates sleep onset, and the nocturnal peak secretion contributes to sleep maintenance in healthy subjects. It has been shown, by measuring plasma melatonin and its urinary metabolite, that almost all patients with SMS display a phase shift in their circadian melatonin rhythm, with the onset of melatonin secretion occurring around 06:00, a peak around 12:00, and an offset around 20:00 without delays in cortisol secretion [9,10,13].

Sleep–wake cycle disturbances could play a significant role in learning deficits, as well as in the frequency and severity of behavioral abnormalities (especially attention disorders), observed in SMS. The behavioral phenotype of SMS is complex and includes maladaptive behaviors, both injuries and tantrums, stereotypic behaviors, and feeding difficulties. These issues may be partly related to cognitive deficits, but they are often much more severe than those observed in patients without SMS but of a comparable developmental level, suggesting that sleep abnormalities may reinforce their behavioral profiles [14]. Sleep disorders are indeed recognized as one of the predictive factors of maladaptive behavior in this population, among other factors such as environmental contingencies, anxiety related to change, and levels of impulsivity [14,15]. To address sleep disorders in SMS, in addition to behavioral techniques, experts have recommended the use of a therapeutic combination of prolonged-release melatonin in the evening at bedtime, to recreate a nocturnal secretion profile, and beta-blockers (β-blockers; adrenergic antagonists) in the morning on waking, to inhibit the abnormal diurnal secretion. Although no clinical trial has been carried out yet, this therapeutic combination appears to reduce the problems associated with circadian rhythms in the adult patients studied [15,16,17,18]. The objective of this study is to describe the clinical, sleep, and chronobiological characteristics of children with SMS under treatment for sleep disorders.

## 2. Materials and Methods

### 2.1. Patients

This was a prospective, single-center study on the clinical, sleep, and chronobiological characteristics of 20 children with SMS, from a cohort followed in the pediatric unit of the *Hôpital Psychiatrique du Vinatier* (GenoPsy Team, Lyon, France). Patients with genetically confirmed SMS, aged between 5 and 13 years old, were included between January 2022 and March 2024. Children older than 13 years old were not included for feasibility and homogeneity reasons; in SMS, behavioral disorders increase significantly at puberty, making cooperation during hospitalization difficult. Furthermore, the age of assessment has a known impact on sleep, the circadian system, and some of the variables measured, such as melatonin.

### 2.2. Study Design

**Figure 1:**
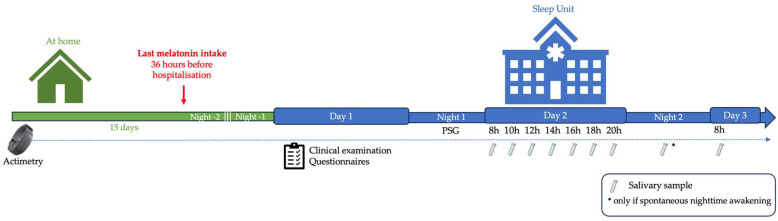
Study design. PSG: polysomnography.

The Sud Est-II Ethics Committee approved the study (19 October 2021, number EudraCT 2020-A03279-30, NCT05116904). Patients who agreed to participate in the study were provided with a home actimetry device, which they wore for 15 days prior to 48 h hospitalization in the Sleep Unit of the *Hôpital Femme Mère Enfant* (Lyon), as well as during the hospital stay. For those who had previously received treatment with melatonin and/or psychostimulants, these treatments were stopped 36 h before hospitalization; sleep characteristics were thus analyzed 48 h after the last melatonin intake. Other treatments (including β-blockers) were maintained to avoid the consequences of abrupt withdrawal. During the 48 h hospitalization period, the children underwent PSG on the first night, as well as other investigations aimed at establishing their clinical, sleep, and chronobiological profiles (Figure 1). Data regarding genetic characteristics, neurocognitive profiles, and therapeutic management were collected from their medical records by the pediatric sleep expert (MC) during the 48 h hospitalization period. The neurocognitive profile was evaluated by a neuropsychologist from the GenoPsy Team during the patients’ follow-up using a Wechsler scale and a Vineland II questionnaire [19,20]. In cases of a neurocognitive assessment performed more than 2 years prior to inclusion, a new assessment was carried out by the same team during the 48 h hospitalization period.

The last melatonin intake occurred 36 h before hospitalization and was resumed in the evening following hospital discharge; the PSG and saliva sampling were thus performed at least 48 h after the last melatonin intake. Treatments other than melatonin (including β-blockers) were maintained to avoid the consequences of abrupt withdrawal. Actimetry was carried out for a period of 15 days prior to hospitalization and continued during the hospital stay in order to assess the effects of melatonin on sleep characteristics.

### 2.3. Clinical Examination and Sleep Questionnaires

At the start of the 48 h hospitalization period, all patients underwent a clinical examination, and questionnaires were completed to assess sleep and behavioral disorders.

Height and weight were obtained for each child and compared to normative data from the French population [21]. The body mass index (BMI = weight/height^2^) was calculated, and the BMI z-score, representing a measure of weight, adjusted for height, sex, and age, relative to a smoothed reference distribution, was computed. Overweight was defined when the BMI z-score was ≥1.6 and ≤1.99 and obesity when the BMI z-score was ≥2 [22].

The Sleep Disturbance Scale for Children (SDSC), validated in French, was completed by parents, adapted to age (version 4 to 16 years). A score > 45/125 is considered pathological. The score was also used to specify the type of disorder encountered according to specific sub-domains: difficulty in initiating and maintaining sleep (>21/35), parasomnias (>17/21), sleep breathing disorders (>12/25), non-restorative sleep (>11/15), and disorders of excessive somnolence (>5/15) [23,24].

Daytime sleepiness was assessed by an adapted version of the Epworth Sleepiness Scale for children and adolescents, validated in French (FSSA) [25], which was completed by the parents. A score above 11 is considered pathological.

The Insomnia Severity Index (ISI), completed by parents, was used to evaluate the quality of nighttime sleep through 7 items scored from 0 to 4 [26]. A final score of 8–14 indicates mild insomnia, while a score of 15–21 suggests moderate insomnia, and a score above 21 indicates severe insomnia.

The Horne and Östberg Morningness–Eveningness Questionnaire is composed of 5 questions to assess an individual’s chronotype (2 to 18 years): definitely of evening type (score 5–8), moderately of evening type (9–11), intermediate type (12–17), moderately of morning type (18–20), and definitely of morning type (21–25). The questionnaire was completed by the parents, with the help of the child when possible.

The 48-item Conners’ Parent Rating Scale (CPRS-48) [27] was used to assess behavioral and attention disorders. This scale is divided into six components (conduct, learning, psychosomatic, impulsivity, anxiety, and hyperactivity), the scores of which are standardized according to age groups. Moderate to severe symptoms are defined with a cut-off above 65 and severe symptoms are defined with a cut-off above 75.

### 2.4. Actimetry

Actimetry was used to analyze sleep quality and obtain chronobiological data. The actimetry device (Actiwatch 2, Philips Respironics, Murrysville, PA, USA, CE certification no. 1104768) was worn at home over a period of about 15 days preceding 48 h hospitalization, to analyze sleep characteristics while patients were under their usual treatment. The actimetry device was also worn during the 48 h hospitalization period in order to assess sleep characteristics without exogenous melatonin.

#### 2.4.1. Sleep Data

Parents completed a sleep diary on each day that sleep was assessed by actimetry. The actimetry devices were attached to the non-dominant wrists of the children and removed only during periods of contact with water (showering, etc.). Children were monitored during the academic year, including one or two weekends. Data for each actigraphy measure were aggregated (averaged) separately for week (scheduled, SC) and weekend (free, FR) nights. Data were analyzed in 1 min epochs and translated into sleep measures by the software Actiware (version 6.1.2), using the scoring procedures described by Acebo et al. (2005) [28]. The scoring interval was defined as 30 min before the reported bedtime in the sleep diary to 30 min after the reported rising time. Data were evaluated at a medium sensitivity threshold. The following actigraphy sleep measures were analyzed: bedtime (as indicated in the diary), sleep start time (defined as the first min of at least three consecutive min of scored sleep after bedtime), sleep end time (defined as the last min of at least five consecutive min of scored sleep just prior to the reported rise time), time spent in bed (TSB), sleep period (SP, defined as the difference between sleep start time and sleep end time), sleep latency (SL, defined as the difference between bedtime and sleep start time), total sleep time (TST, number of minutes scored as sleep in the SP), fragmentation index, sleep efficacy (TST/TSB × 100), sleep efficiency (TST/SP × 100), wakefulness after sleep onset (WASO, defined as SP−TST), and longest continuous sleep episode (LSE, longest uninterrupted sleep episode or interrupted by less than five consecutive minutes of scored wake on actimetry [29]). According to the National Sleep Foundation [30], normal values for sleep duration range between 10 and 13 h per day for children aged 3 to 5 years old and between 9 and 11 h per day for those aged 6 to 13 years old. The following normal values were considered: sleep fragmentation (≤30/h), sleep efficiency (≥85%), and sleep efficacy (≥80%) [31].

#### 2.4.2. Chronobiological Data

The midpoint of sleep was calculated based on actimetry, as well as on sleep questionnaires that reported bedtime, SL, and wake-up time. These data during weekdays and weekend days were used to calculate sleep onset and sleep durations. Then, the midpoint of sleep was calculated during weekdays (MSW) and weekend days (MSF) according to the Munich questionnaire method [32] as sleep onset + sleep duration/2. As no normative data regarding MSW and MSF in children are available, unpublished data obtained from the Elfe cohort study [33] (provided by SP) were used as a reference for the midpoint of sleep. The latter was calculated by including 5 weekdays and 2 weekend days and varied according to age as follows: 2.1 ± 0.4 in children aged 5.5 years old and 2.3 ± 0.4 in children aged 10.5 years.

Based on the methodology used in Mitchell et al. [34] and Yavuz-Kodat et al. [29], the following non-parametric circadian rhythm analysis (NPCRA) parameters were also calculated: intradaily variability (IV), interdaily stability (IS), M10, L5, and relative amplitude (RA). IV provides an estimate of the fragmentation of the 24 h rest–activity rhythm (IV ≈ 0 for a perfect sine wave, IV ≈ 2 for Gaussian noise). Higher IVs would be observed among those who often nap during the daytime and are more frequently awake during the night. IS provides an estimate of how closely the 24 h rest–activity rhythm follows the 24 h light–dark cycle (IS ≈ 0 for Gaussian noise, IS ≈ 1 for perfect stability). A higher IS value indicates good synchronization to light and other environmental cues that regulate the biological clock and therefore a more stable rhythm. M10 reflects the most active period of wakefulness (midpoint of M10). L5, on the other hand, reflects levels of rest during the night: the time of day when L5 occurs (midpoint of L5) indicates whether a person prefers to go to bed earlier or later in the day. RA is the difference between M10 and L5 in the average 24 h pattern, normalized by their sum; higher RA therefore indicates a more robust 24 h rest–activity rhythm, reflecting both relatively lower activity during the night and higher activity when awake.

The normative values in children (mean 11.6 years) [34] are as follows: IS 0.55 ± 0.13, IV 0.76 ± 0.20, M10 12h51, L5 03h21, and RA 0.87 ± 0.07.

### 2.5. Polysomnography

The PSG recordings were obtained using a Morpheus device (Micromed, Roma, Italy). During hospitalization, the children were free to go to bed and wake up whenever they wished in the room.

The PSG included 8 electroencephalograms referenced to the mastoids according to the 10–20 system, 2 electro-oculograms, 1 chin surface electromyography, nasal pressure through cannula, thoracic and abdominal belts, 1 electrocardiogram, and transcutaneous oximetry during the night [35]. Sleep stages, arousals, and respiratory events were scored visually according to standard pediatric criteria [36]. TST, SL, rapid eye movement (REM) latency, sleep efficiency, sleep efficacy, WASO, the fragmentation index (sum of arousal and awakening index per hour), the duration and percentages of stage 1 (N1), stage 2 (N2), stage 3 (N3), and REM sleep, the arousal index, the respiratory arousal index, the obstructive apnea hypopnea index, the index of desaturation < 3%, and minimal saturation were scored by pediatric sleep experts according to the American Academy of Sleep Medicine (AASM 2012 [36]). Age-specific norms for the sleep macrostructure were derived from the study by Scholle et al. [37]. OSA was defined by an obstructive apnea–hypopnea index (OAHI) > 1/h and categorized as mild OSA if OAHI was >1 and <5 /h, moderate OSA if OAHI was ≥5 and <10/h, and severe OSA if OAHI was ≥10/h (12).

### 2.6. Laboratory Assessment

Salivary melatonin and cortisol levels were determined in samples drawn every 2 h for 24 h during the wake periods (a minimum of 3 time points was required to analyze the results) using saliva tablets (Salimetrics, SalivaBio’s Children’s Swab (SCS) System, Carlsbad, CA, USA). The lighting environment was controlled during hospitalization to maintain ambient light levels below 20 lux to prevent any interference with melatonin measurements, as its levels can vary depending on light exposure.

The evaluation of a saliva profile represents a non-invasive approach that allows for the rapid collection of a daytime profile. Each parameter studied (melatonin and cortisol) required the collection of 100 µL of saliva, which was stored in a refrigerator at 4 °C during the patient’s hospitalization and then sent to the laboratory, where centrifugation and aliquoting were performed. The samples were then frozen at −20 °C until the analysis of multiple patients in the same run. To obtain a sufficient volume of saliva for the analysis of the different hormones, the child was asked to keep the saliva collection device in the mouth for 90 s. Melatonin and cortisol levels were determined using liquid chromatography with tandem mass spectrometry (LC-MS/MS). Salivary melatonin levels were categorized as low (≤0.5 pg/mL), moderate (0.5 to 50 pg/mL), and high (≥50 pg/mL), and these were found, respectively, in 20%, 62%, and 17% of children, in accordance with previous studies [38]. Cortisol levels typically peak at around 08:00 a.m. (median 8.4 nmol/L) [39]. In this study, since no published pediatric standard currently provides more than three salivary measurement points within a 24 h period, patients’ results were compared with personal reference data collected in young adults by one of the authors (V.R.). Outlier values (melatonin > 1000 pg/mL or cortisol > 100 nmol/L) were excluded.

### 2.7. Statistical Analyses

Continuous measures were expressed as medians and ranges. Dichotomous and polytomous measures were expressed as numbers and percentages. Comparisons between actimetry periods with and without melatonin treatments were performed using Wilcoxon signed-rank tests for continuous measures. Statistical analysis for the comparison between patients with and without β-blockers could not be performed due to the small number of patients under β-blockers.

Regarding the NPCRA parameters, due to the circular nature of time data and the lack of methodological detail in the reference article [34], L5 midpoint decimal hour values above 20 were adjusted by subtracting 24 h to reflect their temporal proximity to early-night values. This was done to prevent the artificial inflation of the group mean. For the M10 midpoint hours, values under 6 were adjusted by adding 24 h to reflect that these activity midpoints likely occurred late at night and were thus part of the previous day’s active phase. As IS was normally distributed in both conditions (Shapiro–Wilk *p* > 0.6), a paired *t*-test was used. For all other variables that violated normality assumptions, the non-parametric Wilcoxon signed-rank test was applied. The Holm–Bonferroni method was applied to account for multiple comparisons. The statistical significance value for comparisons was set to a *p*-value below 0.05. Statistical analyses were conducted using Jamovi (retrieved from https://www.jamovi.org, Version 2.6.2.).

## 3. Results

### 3.1. Characteristics of the Study Population (Table 1)

The cohort was 55% female, with a median age of 10 years (5 years and 5 months to 13 years and 8 months). Three patients were obese (two class I and one class II), four were overweight, and one was moderately underweight; the weight and BMI z-scores increased with age (r = 0.597 and 0.712, *p* = 0.005 and *p* < 0.001, respectively).

A genetic diagnosis was obtained at a median of 40.5 months (11 to 112 months, n = 20): 75% of patients (15/20) were carriers of a chromosome 17 microdeletion (diagnosed by FISH or CGH) and 25% (5/20) of a RAI1 gene mutation (diagnosed from intellectual disability gene panels). According to the parents, the first sleep disorders appeared at a median of 21 months (0 to 48 months, n = 12).

A prolonged-release melatonin preparation (1 to 20 mg daily) was taken by 95% (19/20) of the patients, in the evening, 30 to 60 min before bedtime (including one in dietary supplement form). Among these patients, one also occasionally used an immediate-release form during nocturnal awakening. Melatonin was started at a median age of 4 years (2 to 13 years). Patients had been treated with melatonin for a median of 5 years (1 day to 9 years). Only 20% (4/20) of patients were under β-blockers (0.8 to 8.7 mg/kg/d), taken in the morning on awakening; for three of these children, β-blockers were taken in association with melatonin. Eight other children had stopped β-blockers in the absence of a clinical improvement, but none reported any side effects leading to the discontinuation of treatment.

Among other prescribed treatments, 55% (11/20) of patients were on psychostimulant (methylphenidate), 2/20 on antipsychotics (risperidone), and 2/20 on antihistamines (alimemazine). One patient was also taking an antidepressant (sertraline).

**Table 1:** Study population characteristics.

Subject Code	Sex, Age (Years)	Weight in kg (±SD) Height in cm (±SD) *	Type of Mutation	Education	Treatment	Intellectual Developmental Disorder **
01	F, 7	18 (−1.5) 109 (−2)	Microdeletion	SNEC	Slenyto^®^ 5 mg Metoprolol 15 mg (0.8 mg/kg/j)	Moderate
02	F, 12	68 (4.5) 152 (1)	*RAI1* mutation	LUSI	Circadin^®^ 20 mg Methylphenidate 15 mg ***	None
03	M, 12	46 (1.5) 145 (0)	*RAI1* mutation	LUSI	Slenyto^®^ 10 mg Acebutolol 400 mg (8.7 mg/kg/j) Methylphenidate 15 mg ***	None
04	M, 12	52 (2) 150 (0.5)	Microdeletion	SNEC	Slenyto^®^ 10 mg Methylphenidate 20 mg ***	Moderate
05	F, 9	34 (2) 127 (−0.5)	*RAI1* mutation	LUSI	Slenyto^®^ 3 mg	None
06	M, 12	68 (4) 161 (2)	Microdeletion	SNEC	Slenyto^®^ 10 mg Methylphenidate 20 mg × 2 *** Risperidone 0.5 mL in the evening	Mild
07	F, 12	49 (2) 150 (0.5)	Microdeletion	SNEC	Slenyto^®^ 10 mg Acebutolol 200 mg (4.1 mg/kg/j) Methylphenidate 30 mg ****	None
08	F, 12	36 (0) 147 (0)	Microdeletion	SNEC	Slenyto^®^ 1 mg Alimemazine 5 drops in the evening *****	Moderate
09	F, 13	53 (1.5) 146 (−1.5)	Microdeletion	SNEC	Slenyto^®^ 6 mg Methylphenidate 20 mg ***	Mild
10	F, 8	20 (−1.5) 117 (−1.5)	Microdeletion	LUSI	Slenyto^®^ 10 mg Methylphenidate 20 mg ******	None
11	M, 11	67 (1) 155 (2.5)	Microdeletion	LUSI	Slenyto^®^ 10 mg	Moderate
12	M, 9	25 (−0.5) 125 (−1)	Microdeletion	SNEC	Acebutolol 200 mg (8.0 mg/kg/j)	Moderate
13	F, 7	16 (−2.5) 107 (−3.5)	Microdeletion	LUSI	Melatonin dietary supplement 7.6 mg	Moderate
14	F, 8	25 (0.5) 121 (−0.5)	Microdeletion	LUSI	Slenyto^®^ 7 mg	None
15	F, 12	39 (−1) 149 (−1)	Microdeletion	SNEC	Slenyto^®^ 5 mg Methylphenidate 10 mg × 2 ****	Moderate
16	M, 6	18.4 (1) 112 (−2)	*RAI1* mutation	Regular school	Slenyto^®^ 10 mg Methylphenidate 20 mg **** Sertraline 25 mg	None
17	M, 11	29 (−1) 135 (−1)	*RAI1* mutation	LUSI	Slenyto^®^ 5 mg Methylphenidate 10 mg × 2 ***	None
18	M, 7	20 (0) 118 (0)	Microdeletion	Regular school	Slenyto^®^ 10 mg Methylphenidate 10 mg × 2 ***	None
19	F, 9	26 (−1) 143 (1.5)	Microdeletion	SNEC	Slenyto^®^ 6 mg Methylphenidate 20 mg, 10 mg **** Risperidone 0.5 mL Alimemazine 20 drops *****	None
20	M, 5	17 (−2) 110 (−1)	Microdeletion	Regular school (awaiting SNEC)	Slenyto^®^ 10 mg	NA

F: female. LUSI: Localized Unit for School Inclusion. M: male. NA: not available. SD: standard deviation. SNEC: Special Needs Education Center. * The SD reported for each individual value of weight and height represents the deviation from the normative data of the French population according to age [21]. ** Intellectual developmental disorder was evaluated by a neuropsychologist using the Wechsler scale and Vineland II questionnaire and could be considered as mild, moderate, or non-existent (none). *** Medikinet^®^. **** Ritaline^®^. ***** Theralène^®^. ****** Quazym^®^.

### 3.2. Subjective Sleep and Behavioral Characteristics Under Treatment for Sleep Disorders (Table 2)

The children had different bedtimes on weekends (21:30) compared to weekdays (20:37), but their wake-up times were very consistent (06:07). Ten children reported a nap at least once a week. Midsleep was, respectively, 01:30 during weekdays and 01:55 during weekend days (*p* < 0.001), both earlier than normative values.

The SDSC total score showed significant sleep disorders in 70% of patients (14/20), sleep breathing disorders in 20% (4/20), difficulties in initiating and maintaining sleep in 50% (10/20), and disorders of excessive somnolence in 40% (8/20). Pathological daytime sleepiness was also found on the FSSA in 65% of patients (13/20), as well as mild (8/20), moderate (9/20), and severe insomnia (1/20) according to the ISI in 90% of patients (18/20). Finally, the Horne and Ostberg chronotypes confirmed the morning type in 70% of patients (moderately and definitely, 14/20, 10 of whom had a score > 21) and an intermediate type in the remaining 30% (6/20). No patient had an evening chronotype.

**Figure 2:**
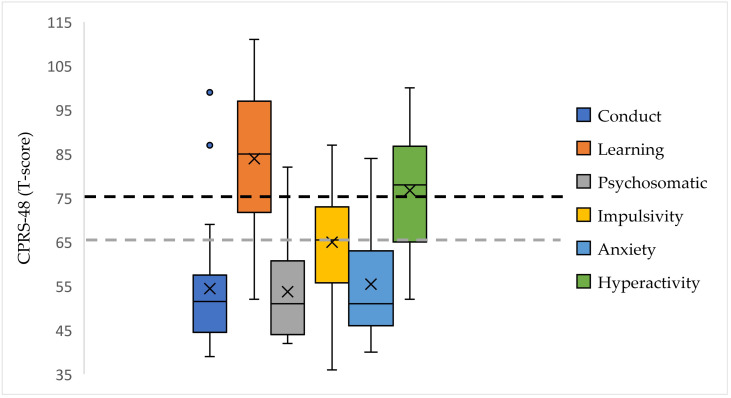
Results from the 48-item Conners’ Parent Rating Scale (CPRS-48). The scores for the 6 components of the CPRS-48 are represented graphically using box plots. The pathological cut-off for moderate to severe (>65) is shown by the grey dotted line and the cut-off for severe (>75) by the black dotted line.

The CPRS-48 revealed the presence of learning problems in 90% of the patients (18/20), which were severe in 72% (13/18). Hyperactivity was present in 80% of patients (16/20), impulsivity in 50% (10/20), and anxiety disorders in 20% (4/20), and psychosomatic problems were less common (15%, 3/20; Figure 2).

When compared to children without β-blockers, those on β-blockers tended to go to bed slightly later (21:00 vs. 20:37), wake up earlier (05:37 vs. 06:15), have longer nighttime awakenings (60 vs. 15 min), experience more complaints of insomnia (ISI 17.5 vs. 14.0), and report more daytime sleepiness (FSSA 13 vs. 10). Children on beta blockers had a midpoint of sleep at 01:15 on weekdays and 01:52 on weekends, while children without beta blockers had a midpoint of sleep at 01:30 on weekdays and 02:00 on weekends, reflecting no social jetlag. On the hyperactivity component of the CPRS-48, the four children on β-blockers had a score of 71.5 (60.0–99.0), compared to 80.0 (52.0–100.0) for children without β-blockers.

**Table 2:** Sleep questionnaire results for patients with and without β-blockers.

	Total Population (n = 20)	Patients without β-Blockers (n = 16)	Patients with β-Blockers (n = 4)
SDSC			
Bedtime (weekdays/weekends)	20:37 (19:30–22:30)/ 21:30 (19:30–22:30)	20:37 (19:30–21:30)/ 21:30 (20:00–22:30)	21:00 (19:45–22:30)/ 22:15 (19:30–22:30)
Sleep end time (weekdays/weekends)	06:07 (03:00–07:30)/ 06:07 (03:00–08:30)	06:15 (03:00–07:30)/ 06:22 (03:00–08:30)	05:37 (05:00–07:00)/ 05:37 (05:00–08:15)
Naps in minutes (weekdays/weekends)	0 (0–90)/15 (0–120)	0 (0–60)/15 (0–60)	0 (0–90)/30 (0–120)
Wakefefulness after sleep onset (weekdays/weekends)	22 (0–120)/30 (0–120)	15 (0–120)/ 30 (0–120)	60 (0.2–120)/ 60 (0.2–90)
Number of night awakenings	2 (0–5)	1.5 (0–5)	2 (1–2)
Sleep disorders (/125)	46.5 (31.0–57.0)	47.5 (31.0–57.0)	44.0 (39.0–48.0)
Difficulty in initiating and maintaining sleep (/35)	18.5 (11.0–31.0)	19.0 (11.0–31.0)	17.5 (11.0–20.0)
Parasomnias (/35)	11.0 (7.0–18.0)	11.5 (7.0–18.0)	10.0 (7.0–11.0)
Sleep breathing disorders (/25)	7.0 (5.0–12.0)	7.0 (5.0–12.0)	7.0 (6.0–12.0)
Non-restorative sleep (/15)	4.0 (2.0–7.0)	4.0 (2.0–7.0)	3.5 (2.0–6.0)
Disorders of excessive somnolence	6.0 (3.0–8.0)	6.0 (3.0–8.0)	6.5 (3.0–8.0)
MSW	01:30 (23:52–02:45)	01:30 (23:52–02:30)	01:15 (00:30–02:45)
MSF	01:52 (00:00–03:22)	02:00 (00:00–03:00)	01:52 (00:22–03:22)
FSSA	11.5 (2.0–17.0)	10.5 (2.0–17.0)	13.0 (6.0–14.0)
ISI	14.5 (4.0–26.0)	14.0 (7.0–26.0)	17.5 (4.0–20.0)
Horne and Ostberg	20.0 (13.0–25.0)	21.0 (13.0–25.0)	17.5 (14.0–21.0)

Data are presented as median (range). FSSA: Epworth Sleepiness Scale for children and adolescents, validated in French. ISI: insomnia severity index. MSF: midpoint of sleep during weekend days. MSW: midpoint of sleep during weekdays. SDSC: Sleep Disturbance Scale for Children.

### 3.3. Objective Sleep Characteristics on Actimetry with and Without Melatonin (Table 3)

The effect of melatonin treatment was analyzed in 13 patients who underwent actimetry recording both with and without melatonin. Three patients did not tolerate wearing the watch at home, and three others had a recording in only one condition. Another patient had no exogenous melatonin and was not included in this analysis. Three of the 13 patients were also taking β-blockers. A median of 11 nights (4–14) were recorded and interpretable for each patient.

When comparing periods recorded with melatonin to those without melatonin, a significant benefit of exogenous melatonin was found regarding the mean duration of WASO (1.4 vs. 2.3 min, *p* = 0.040), wake-up time (06:50 vs. 06:11, *p* = 0.004), and LSE (398 vs. 317 min, *p* = 0.040), but only a tendency toward increased nighttime TST (460 vs. 452 min, *p* = 0.068), higher sleep efficacy (86.1 vs. 80.1%, *p* = 0.191), and a lower WASO duration (60.0 vs. 80.5 min, *p* = 0.080) was observed. Other sleep parameters did not differ significantly according to the presence of exogenous melatonin.

Following the administration of exogenous melatonin, patients exhibited normal sleep onset latency (<30 min) at an age-appropriate bedtime (20:55; range 19:50–22:22). Sleep efficiency, efficacy, and fragmentation were within the expected ranges for their age, but TST remained shorter. When comparing individual values to reference values [29,31], after melatonin supplementation, two patients had normal TST (none before treatment), seven had normal sleep efficiency (four before treatment), and 10 had a normal fragmentation index (eight before treatment).

**Table 3:** Actimetry results for periods with and without melatonin treatment.

	Total Population (n = 13)	Periods without Melatonin	Periods with Melatonin	*p* (Wilcoxon Test)
Bedtime	21:08 (19:50–00:24)	21:13 (19:31–22:42)	20:55 (19:50–22:22)	0.273
Wake-up time	06:48 (01:30–09:26)	06:11 (05:08–07:53)	06:50 (06:08–08:12)	0.004
Sleep start time	21:28 (19:16–01:05)	21:20 (19:46–22:47)	21:28 (20:17–22:36)	1.000
Sleep end	06:20 (01:20–08:50)	06:02 (04:45–07:14)	06:27 (05:19–08:01)	0.273
SP	08:53 (04:31–10:53)	08:43 (05:58–10:43)	08:57 (06:23–10:20)	0.588
SL (min)	15.5 (0–98.0)	8.0 (0.5–44.0)	17.5 (5.5–29.5)	0.080
Sleep efficiency (%)	79.9 (48.3–98.6)	75.4 (65.4–96.7)	79.5 (61.7–87.8)	0.735
Sleep efficacy (%)	88.4 (59.3–99.9)	80.1 (71.1–97.2)	86.1 (69.6–95.1)	0.191
WASO (min)	59.0 (0.5–250.0)	80.5 (15.5–180.0)	60.0 (18.3–195.0)	0.080
Number of WASO	37 (1–93)	31 (14 –60)	36 (18–53)	1.000
Mean duration of WASO (min)	1.6 (0.5–11.9)	2.3 (1.1–6.2)	1.4 (0.8–7.9)	0.040
TST (nighttime, min)	454 (244–629)	452 (280–530)	460 (340–560)	0.068
Fragmentation (%)	23.6 (0.5–53.1)	26.9 (2.0–41.5)	24.8 (8.2–39.7)	0.414
LSE (min)	384 (107–693)	317 (227–559)	398 (291–610)	0.040
MSW	01:47 (00:36–03:35)	01:02 (23:53–02:09)	01:45 (00:55–01:44)	0.162
MSF	02:06 (01:11–02:44)	NA *	NA *	NA *
L5	02:00 (00:25–10:52)	01:20 (00:25–23:55)	01:40 (00:11–10:52)	0.677
M10	12:43 (02:26–22:02)	13:35 (09:33–16:41)	12:47 (01:43–22:02)	0.970
IS	0.46 ± 0.15	0.67 ± 0.18	0.51 ± 0.16	0.057
IV	0.60 ± 0.13	0.75 ± 0.29	0.62 ± 0.14	0.092
RA	0.80 ± 0.19	0.87 ± 0.09	0.82 ± 0.18	0.423

IS: interdaily stability. IV: intradaily variability. LSE: longest continuous sleep episode. MSF: midpoint of sleep during weekend days. MSW: midpoint of sleep during weekdays. NA: not available. RA: relative amplitude. SL: sleep latency. SP: sleep period. TST: total sleep time. WASO: wakefulness after sleep onset. The analysis of the total population (n = 13) represented 155 nights, corresponding to 127 nights with melatonin and 28 nights without melatonin. * Comparison between weekend nights with and without melatonin was not possible as all children were hospitalized during the week, and nights without melatonin were recorded during hospitalization.

### 3.4. Chronobiological Data (Table 3)

The MSW under melatonin tended to be later (01:45) than during periods without melatonin (01:02, *p* = 0.162), and both were earlier than normative values. In the total population, L5 occurred more than an hour earlier (02:00) compared to age norms, while M10 was within normative values (12:43); no significant effect of exogenous melatonin was observed. IV and IS were within age norms (0.60 and 0.46) in the total population; a trend toward increased IV and IS was observed during periods without melatonin (0.75 vs. 0.62 and 0.67 vs. 0.51, respectively).

### 3.5. Objective Sleep Characteristics on Polysomnography (Table 4)

Only one patient (patient 17) did not undergo PSG due to major behavioral disturbances that prevented the installation of the various sensors required for the examination.

During hospitalization, patients fell asleep at 21:30 (19:42 to 23:40), with a short median latency of 7.9 min (1.7 to 174.4 min); 15 patients had normal latency (<20 min) and only four patients had sleep onset latency > 30 min. Sleep was fragmented in 74% (14/19) of patients. When compared to the norms expected for this age group, TST (410 min) and sleep efficacy (70.3%) were lower and the sleep fragmentation index (21.5) was higher, probably due to long nocturnal awakenings: all patients had WASO durations > 10% of their SP, and 78.9% (15/19) had prolonged WASO lasting more than one hour continuously, with the longest WASO occurring at a median of 04:00 (23:42 to 05:54).

Among all patients, 80% (16/20) of them took a nap during the day following the PSG recording, according to their habits; three of them had two to three naps during the day. The median duration of naps was 47 min (9 to 163 min) and they started at 13:27 (10:49 to 17:47; four in the late morning and 16 in the afternoon). The 24 h TST was lower than the expected norms according to age for all patients, with a median duration of 471 min (277 to 532 min). There was no significant difference in TST depending on whether or not a nap was present (410 min versus 426 min, *p* = 0.29).

The dominant posterior rhythm was well visualized on eye closure, and all patients showed normal sleep patterns (sleep spindles and K-complexes). The proportion of different sleep stages was respected overall, although there was the small overrepresentation of N1, possibly related to a first night effect. It should be noted that, although the median time spent in REM (18.8% TST) was within the expected norms, 47% of patients (9/19) had a percentage of REM lower than 17%. Nycthemeral organization was normal in all patients.

Electroencephalographic abnormalities, which were unknown and non-symptomatic, were observed in five patients: centrotemporal (patients 01 and 18), occipital (patient 03), central (patient 11), and frontal (patient 15). None had a history of seizures.

From a respiratory point of view, 50% of patients (10/20) had OSA: eight mild and two severe (patients 04 and 06, both aged 12 and overweight, with an OAHI of 17.6 and 29.7 per hour, respectively). The two patients with severe OSA complained of snoring and presented repercussions on saturation (minimum saturation at 85 and 83%, time spent < 90% at 0.6 and 0.5% TST, respectively) and sleep fragmentation (index at 21.5 and 53.6 per hour, respectively), but physiological capnia values. They had never been clinically screened and therefore had no previous nocturnal recordings. They were referred for specialist OSA management. For the other eight patients, there was no respiratory effort, no recruitment of accessory muscles, and no thoracoabdominal asynchrony. None of the patients had periodic leg movements.

Patients on β-blockers (n = 4) tended to fall asleep later than patients without β-blockers (22:10 vs. 21:24) and to have a longer SL (55.9 vs. 7.9 min) and a higher number of WASO episodes (41 vs. 24). They also tended to have a lower nighttime TST (398 versus 424 min). Children on beta blockers had a midpoint of sleep at 01:20 on weekdays and 01:35 on weekends, while children without beta blockers had a midpoint of sleep at 01:13 on weekdays and 01:36 on weekends.

**Table 4:** Polysomnography (in patients with melatonin-free recordings).

	Total Population (n = 19)	Patients without β-Blockers (n = 15)	Patients with β-Blockers (n = 4)
Sleep onset	21:30 (19:42–23:40)	21:24 (19:42–23:27)	22:10 (22:02–23:40)
Sleep offset	06:37 (04:00–09:05)	06:35 (04:03–09:02)	07:27 (06:29–09:05)
Sleep efficacy (%)	70.3 (52.1–86.8)	65.0 (50.9–84.2)	61.8 (50.0–73.0)
TSB (h)	09:46 (06:34–12:29)	09:46 (06:34–11:31)	10:17 (08:49–12:29)
TST (nighttime, min)	410 (232–490)	424 (232–473)	398 (264–490)
TST (over 24 h, min)	471 (277–532)	471 (277–532)	435 (313–490)
MSW	02:03 (00:49–04:22)	01:59 (00:49–03:38)	02:48 (02:16–04:22)
WASO (min)	166.5 (67.5–293.5)	170.5 (67.5–293.5)	159.5 (74.5–240.5)
Number of WASO	30 (6–53)	24 (6–52)	41 (28–53)
SL (min)	7.9 (1.7–174.4)	7.9 (1.7–36.9)	55.9 (5.4–174.4)
REM onset latency (min)	145.5 (54.4–353.0)	145.5 (54.5–353.0)	154.0 (138.0–176.5)
Time spent in REM (% TST)	18.8 (7.4–27.7)	16.9 (7.4–27.7)	20.5 (13.6–22.5)
Time spent in N1 (% TST)	10.8 (0.9–22.5)	10.3 (0.9–22.5)	13.7 (10.2–19.5)
Time spent in N2 (% TST)	42.1 (32.7–60.0)	42.1 (35.2–60.0)	43.4 (32.7–52.9)
Time spent in N3 (% TST)	27.4 (11.6–33.5)	28.9 (18.5–33.5)	27.2 (11.6–27.6)
OAHI (/h)	1.7 (0–29.7)	1.4 (0–29.7)	2.2 (0.3–2.9)
Desaturation index ≥ 3% (/h)	2.9 (0.1–17.8)	3.1 (0.1–17.8)	1.4 (0.2–4.8)
Arousal index (/h)	18.3 (4.9–47.0)	18.3 (4.9–47.0)	19.3 (12.2–26.8)
Fragmentation index (/h)	21.5 (6.0–53.6)	21.5 (6.0–53.6)	25.4 (18.6–33.5)
Respiratory arousal index (/h)	2.1 (0.3–30.5)	1.7 (0.3–30.5)	3.8 (0.4–6.9)
Median saturation (TST, %)	96.2 (94.6–97.8)	96.2 (94.6–97.7)	97.4 (95.6–97.8)
Minimal saturation (TST, %)	91.0 (83.0–95.3)	91.0 (83.0–94.0)	93.2 (87.0–95.0)
Maximal CO_2_ value (mmHg)	45.9 (40.5–51.1)	45.9 (43.5–51.1)	46.3 (40.5–49.7)
Medium CO_2_ value (mmHg)	41.8 (37.7–48.5)	41.8 (37.7–48.5)	41.2 (38.2–43.8)
Average heart rate (bpm)	74.5 (66.9–88.8)	78.3 (66.9–88.8)	71.5 (71.1–72.3)

CO_2_: carbon dioxide. MSW: midpoint of sleep during weekdays. REM: rapid eye movement. SL: sleep latency. OAHI: obstructive apnea hypopnea index. TSB: time spent in bed. TST: total sleep time. WASO: wakefulness after sleep onset.

### 3.6. Hormonal Profiles of Patients with and Without β-Blockers (Figure 3)

Salivary melatonin levels were determined in 17 patients; three patients were excluded due to insufficient saliva collection (patient 04 only had two values and patient 19 had none) or outlier values (patient 20: median 1397 pg/mL, 207–8979 pg/mL, peak at 10:25 a.m.). Cortisol levels were determined in 19 patients (patient 19 was excluded due to technical difficulties).

**Figure 3:**
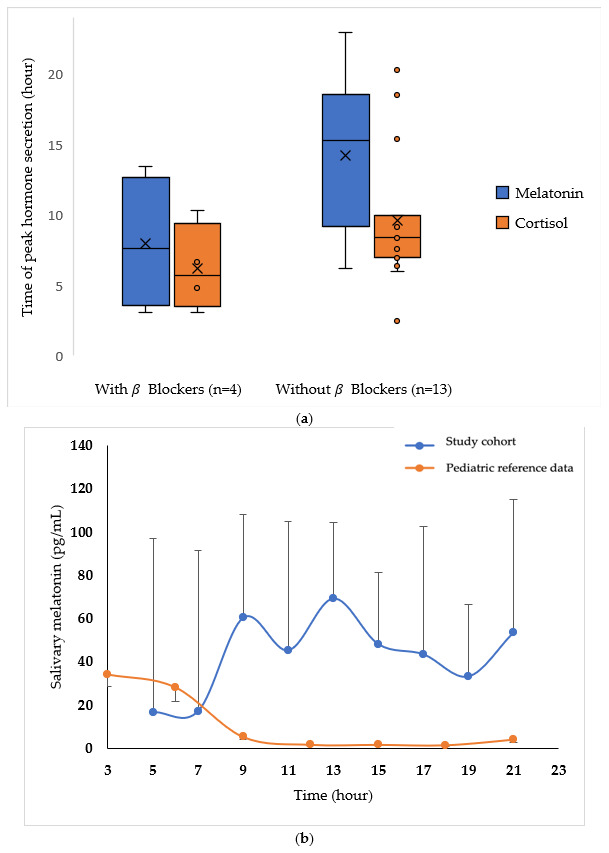

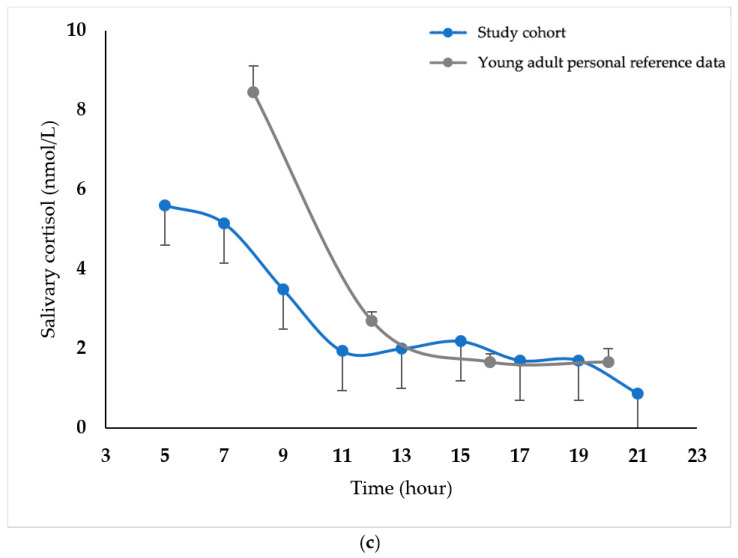
Salivary melatonin and cortisol profiles in children with Smith–Magenis syndrome. (**a**) The time of peak secretion in patients with and without beta blockers. (**b**) The 24 h salivary melatonin profiles in patients compared with healthy children (mean 9 years, range 5 to 12 years) [38]. Each point represents the median value over a 2 h interval (in pg/mL) and is shown with the standard deviation. (**c**) The 24 h variation in salivary cortisol compared to personal reference data in young adults. Each point represents the median value over a 2 h interval (in nmol/L) and is shown with the standard deviation. Detailed data are presented in Appendix A.

#### 3.6.1. Melatonin

A median of nine salivary time points over 24 h (3–13) was analyzed for each patient to establish a secretion profile. Salivary melatonin peak levels occurred during the day at 11:57 (03:05 to 13:26), compared to a physiological peak at 03:00.

In patients on β-blockers, the time of the peak occurred earlier in the day (07:38, range 03:05 to 13:26) compared to patients without β-blockers (15:16, 06:12 to 22:57). Among patients on β-blockers, patients 03 and 07 had a classic dosage (8.7 and 4.1 mg/kg/d, respectively) and a nocturnal peak (04:59 and 03:05, respectively). Patient 01, who had an unusual molecule (metoprolol) and a very low dosage (0.8 mg/kg/d), had a diurnal peak of secretion (13:26). Finally, patient 12, who had a classic dosage (7.9 mg/kg/d) but two nocturnal samples that could not be used due to a lack of saliva, had peak secretion in the first morning sample at 10:18, but a higher nocturnal peak preceding this sample could not be ruled out.

Melatonin levels were high in this cohort, with a median 24 h level of 51.10 pg/mL (3.00 to 297.25), reaching a median peak value of 169.50 pg/mL (5.60 to 831.40), with no obvious difference between patients with and without β-blockers (median levels with β-blockers of 51.50 pg/mL and 49.80 pg/mL and peak levels of 131.80 pg/mL and 343.40 pg/mL, respectively). Overall, 71% of patients (12/17) had peak secretion above 50 pg/mL. Over 24 h, no patient was a low secretor (<0.5 pg/mL), seven (patients 05, 06, 07, 09, 11, 12, and 18) were medium secretors (0.5 to 50 pg/mL), and the remaining 10 were high secretors. The only patient without exogenous melatonin supplementation (patient 12) had a median melatonin level of 3.00 pg/mL, with a peak of 10.90 pg/mL. Of note, the median melatonin levels did not differ significantly between children aged under 10 years old (131.0 pg/mL, 10.9 to 551.0 pg/mL) and those aged at least 10 years old (291.0 pg/mL, 5.6 to 831.0 pg/mL, *p* = 0.389).

#### 3.6.2. Cortisol

Regarding cortisol profiles, a median of 9.0 salivary points (6–13) was analyzed for each patient. The median peak cortisol level reached 8.29 nmol/L (1.57–28.78).

The secretion rhythm was visually highly pulsatile, with a median secretion peak at 08:22 (02:28–20:18); it was 05:44 in patients with β-blockers and 08:26 in patients without. This peak was shifted to the end of the day (20:18) in patient 17, who also had a marked behavioral disorder with regular tantrums during the day.

## 4. Discussion

This prospective study assessed the clinical, sleep, and chronobiological characteristics of 20 children with Smith–Magenis Syndrome (SMS) undergoing treatment for sleep disorders. Despite treatment, two thirds of the patients reported sleep disorders and excessive daytime sleepiness. Objective measures of sleep duration remained poor for most patients, and sleep quality was also impaired in some of them. The findings herein underline the limited effectiveness of the current treatment for SMS and identifies several important considerations that need to be discussed.

SMS is a complex disorder of genetic origin, the pathognomonic feature of which is a shift in melatonin secretion [9,10,40]. This shift was observed in the present cohort, with peak secretion around noon, but was not associated with a shift in cortisol peak secretion. The preservation of physiological profiles for cortisol, GH, and prolactin [9] does not corroborate the hypothesis of a global alteration in the circadian system (which would be responsible for a shift in all circadian parameters), but rather a very specific disorder of melatonin secretion. Furthermore, the short sleep latency (8 min), early midpoint of sleep (about 30 min earlier than the 02:05 for 5.5 year olds and 02:30 for 10.5 year olds obtained from the Elfe cohort, unpublished data), and early wake-up time (06:11) observed on actimetry (even with exogenous melatonin) and PSG (even under β-blockers) are suggestive of a phase advance in the melatonin secretion rhythm. The clinical consequences of this advance in the phase of melatonin secretion are difficulties in maintaining sleep, associated with significant daytime sleepiness, reflected by clinical complaints persisting even after treatment.

The current recommendations for circadian sleep disorders in SMS involve prescribing melatonin in the evening to compensate for the absence of nocturnal secretion, combined with a β1-adrenergic antagonist in the morning to block the abnormal diurnal peak in endogenous melatonin [13]. However, there have been no randomized therapeutic trials to confirm the effect of this combination, or even studies of its long-term efficacy. The only randomized controlled trial studying the effect of Slenyto^®^ in SMS is based on the study by Gringras et al. [41], which reported an increase in TST and a decrease in sleep onset latency in a cohort of children mainly with autism spectrum disorder (ASD; four children with SMS in a cohort of 125 patients with ASD), without distinguishing the effect of the treatment specifically in this subgroup, probably because of the small number of patients with SMS.

Based on the actimetry findings herein, exogenous melatonin does not appear to greatly improve sleep quantities in children with SMS. Although awakenings were of a shorter duration, LSE (a recent sleep quality marker [29]) increased, and the wake-up time occurred later under melatonin, while sleep efficacy and TST were not significantly improved. This partial effect might explain the high dose of exogenous melatonin prescribed herein (median 7.6 mg). However, the intake of high doses of melatonin may lead to receptor desensitization, as reported by experimental studies showing that melatonin can induce receptor regulation (desensitization and internalization), particularly MT2 receptors [42,43]. Pharmacodynamics studies are lacking in this setting, and, given that the long-term effects and the risk of tolerance/desensitization in the pediatric population are insufficiently studied, caution should be applied when increasing melatonin doses [44]. Importantly, the salivary melatonin levels were very high more than 48 h after the last treatment intake. This effect was already reported by Spruyt et al. [45]. The authors underlined the importance of monitoring melatonin levels in SMS patients, particularly in children. Chik et al. also demonstrated that the salivary melatonin levels were higher in SMS patients receiving exogenous melatonin therapy, even in combination with β-blockers [46]. This is also supported by the fact that the only child in our cohort without exogenous melatonin had physiological salivary melatonin levels. The half-life of Slentyo^®^ is 4.4 to 4.9 h in saliva, longer than immediate-release forms due to very slow absorption, with a flip-flop effect that could be responsible for accumulation during daily use. For example, with 5 mg of Slenyto^®^, the expected salivary levels 20 h after administration would be 25 to 75 pg/mL (higher if taken with food) (European Medicines Agency—Public Assessment Report—EMA/556280/2018). In addition, there may also be increased endogenous melatonin production after the administration of exogenous melatonin, although this mechanism has not yet been thoroughly studied [47]. Thus, these high levels after treatment discontinuation could be indicative of an iatrogenic effect of melatonin supplementation. Indeed, melatonin metabolism is mainly hepatic, via CYP1A2. Large interindividual variations (10- to 200-fold) in CYP1A2 activity are found between individuals, explaining the differences in melatonin’s half-life [48]. In slow metabolizers, who exhibit decreased CYP1A2 enzyme activity, exogenous melatonin can lead to an increase in circulating melatonin levels [49]. Slow metabolizers would therefore respond transiently to exogenous melatonin supplementation, with sleep complaints reappearing after a few weeks of treatment. These slow metabolizers may be more common among those with developmental disorders, which could explain the accumulation of melatonin levels over time with greater dosages prescribed [49,50]. These high levels could therefore be partly iatrogenic, reflecting not only abnormal endogenous secretion but also exogenous melatonin accumulated over time in patients receiving very supraphysiological doses and/or with slow melatonin metabolism. Thus, given the great interindividual variability in endogenous melatonin levels in children, combined with the many factors influencing the response to exogenous melatonin supplementation (sex, age, hepatic metabolism, other treatments, etc.) and the fact that behavioral disorders in SMS appear to be linked in part to the shift in melatonin secretion [9,15], the careful administration and titration of melatonin for personalized medicine should be considered [45,51]. Such monitoring, not yet proposed in current practice, could enable treatment to be better adapted to the patient’s profile to improve sleep quality without aggravating behavioral problems and daytime sleepiness. In the case of elevated melatonin metabolite levels, the dosage of exogenous melatonin should be reduced, a CYP1A2 inducer could be proposed (such as omeprazol or capsules containing cabbage, broccoli, cumin, and turmeric concentrates in the morning), or the melatonin administration schedule could be modified (skip doses at regular intervals).

In healthy subjects, melatonin synthesis is activated by the stimulation of β1-adrenergic receptors in the pineal gland. Melatonin secretion is thus blocked by the use of β1-adrenergic receptor antagonists such as β-blockers. Given in the morning on awakening, and with a duration of action of 3–4 h, they inhibit the abnormal diurnal secretion peak. The effect of antagonist treatment and its action on endogenous melatonin secretion has rarely been studied in SMS children since the initial work of De Leersnyder et al. on nine patients aged 4 to 17 years old [17]. Its effect on sleep quality in this population has, to our knowledge, never been studied, even though insomnia is one of the most well-known side effects of this treatment [52]. In the present cohort, although a statistical analysis could not be carried out due to the small sample size, patients taking β-blockers appeared to have poorer sleep quality, with a tendency toward increased sleep onset latency, later sleep onset, more nocturnal awakenings, and a reduction in TST (both at nighttime and over 24 h). This was the case despite a melatonin hormonal profile that seemed to be effectively restored by the action of β-blockers, with the inhibition of the diurnal peak in patients with a classic dosage. Moreover, children did not seem to perceive any obvious benefit for daytime sleepiness symptoms, as depicted by the non-improved FSSA score compared to patients without β-blockers. However, a small tendency toward decreased hyperactivity symptoms was observed. The low number of patients on β-blockers could be an indirect indicator of the only partial effect of the treatment, since 60% of the cohort had already used β-blockers, and only 20% continued to do so during the study. Of note, the long-term effect of β-blockers on melatonin metabolism has never been studied. In addition, β-blockers can also modulate the response of the hypothalamic–pituitary–adrenal axis to stress, an interesting potential beneficial effect in SMS children, who are particularly sensitive to change [15]. This effect may partly explain the relatively lower dispersion in cortisol peak times observed herein in children under β-blockers, who were exposed to various stressors (related to hospitalization, regular saliva sampling, etc.), similarly to children without β-blockers. However, this lower dispersion could also be related to the small sample size. Co-medication is not uncommon in patients with neurodevelopmental disorders; unfortunately, the sample sizes were too small to examine the effects of other drugs in the present cohort. Psychostimulants, for instance, were prescribed to more than half of the patients herein, 90% of whom presented with a pathological CPRS-48 score; their efficacy, however, has not yet been demonstrated [53]. This high rate of psychostimulant prescription could reflect attentional difficulties linked to the high rate of pathological daytime sleepiness (65% of the present population), itself related to the shift in melatonin secretion and/or the possible iatrogenic accumulation of exogenous melatonin.

Other treatments currently exist, such as oral melatonin receptor agonists, but are less widely used. Tasimelteon [54] has been developed as a chronobiotic agent for the treatment of sleep–wake circadian rhythm disorders. A randomized, double-blind, controlled trial in 25 individuals with SMS demonstrated its efficacy in improving sleep quality and total sleep time but reported side effects (headaches, nightmares, upper respiratory tract infections, and urinary tract infections). Moreover, in animal models, concerns about fertility, development, and carcinogenesis have been raised. Other agonists, such as ramelteon or agomelatine, could be used, but their benefit–risk profiles appear less favorable than that of exogenous melatonin supplementation [55].

Finally, non-pharmacological approaches could be of interest in SMS in order to block abnormal daytime secretion without worsening patients’ sleep quality. Light sensitivity seems to exist in SMS, as patients have a regular (and not free-running) 24 h melatonin secretion rhythm. However, this sensitivity is impaired as daylight does not seem to inhibit abnormal daytime secretion. Only one study [56] has investigated the effect of exposure to intense light at the end of the day in a 6-year-old child with SMS. This normalized the phase of body temperature secretion (usually 3 h ahead of schedule) and also resulted in later sleep onset, fewer nocturnal awakenings, and lower nocturnal motor activity. This case study has not been repeated on a larger scale and did not include data on melatonin secretion before and after the procedure. A better understanding of the pathophysiology of SMS could lead to more appropriate and controlled therapeutic solutions.

The present study has limitations, with the main ones being the small sample size due to the rarity of SMS and the real-life nature of the study. The absence of standardized treatment led to variability in treatment combinations and doses, limiting the possibility to evaluate the effects of each treatment separately. Moreover, since salivary samples were only drawn during wake periods, so as not to bias the PSG results, the analysis of the hormonal secretion profiles relied on a higher number of diurnal samples. Finally, no data relating to puberty status were collected, preventing us from analyzing the potential effect on sleep parameters; nevertheless, melatonin levels did not appear to differ significantly according to age.

## 5. Conclusions

Although objective sleep recordings support the beneficial effect of prolonged-release melatonin supplementation in reducing nocturnal arousals, this intervention alone appears insufficient to ensure satisfactory sleep quantity, quality, and circadian alignment as reflected by midpoint values. Moreover, the iatrogenic accumulation of exogenous melatonin is possible, due to the very supraphysiological doses received by patients and the possibility of a high proportion of slow metabolizers among these patients, with a potential aggravating role of this accumulation in the behavioral difficulties already present in children with SMS. This hypothesis is supported by the fact that the only patient in the cohort without exogenous melatonin supplementation had normal salivary melatonin levels. While the biological interest of β-blockers seems convincing, their effects on wakefulness and behavior remain to be proven, and their possible negative consequences for sleep deserve to be further studied. These findings should lead to more cautious, individualized prescribing, based on melatonin monitoring during treatment. The use of non-medical techniques such as light therapy to try to better synchronize melatonin secretion could be considered.

## Data Availability

The data can be obtained from the corresponding author upon request.

## Abbreviations

The following abbreviations are used in this manuscript:

AASM	American Academy of Sleep Medicine
BMI	Body Mass Index
CGH	Comparative Genomic Hybridization
CO_2_	Carbon dioxide
CPRS-48	48-Item Conners’ Parent Rating Scale
FISH	Fluorescence In Situ Hybridization
FSSA	Epworth Sleepiness Scale for Children and Adolescents Validated in French
IS	Interdaily Stability
ISI	Insomnia Severity Index
IV	Intradaily Variability
LC-MS/MS	Liquid Chromatography with Tandem Mass Spectrometry
LUSI	Localized Unit for School Inclusion
LSE	Longest Continuous Sleep Episode
MSF	Midpoint of Sleep During Weekdays
MSW	Midpoint of Sleep During Weekend Days
N1	Stage 1
N2	Stage 2
N3	Stage 3
NPCRA	Non-Parametric Circadian Rhythm Analysis
OAHI	Obstructive Apnea Hypopnea Index
OSA	Obstructive Sleep Apnea
PSG	Polysomnography
RA	Relative Amplitude
RAI1	Retinoic Acid Induced 1
REM	Rapid Eye Movement
SDSC	Sleep Disturbance Scale for Children
SL	Sleep Latency
SMS	Smith–Magenis Syndrome
SNEC	Special Needs Education Center
SP	Sleep Period
TSB	Time Spent in Bed
TST	Total Sleep Time
WASO	Wakefulness After Sleep Onset

## Appendix A

In order to establish the salivary melatonin and cortisol secretion profiles of patients, we grouped all melatonin and cortisol values from the cohort into two-hour intervals. The number of data points for each time interval is shown in the tables below.

**Table A1:** Melatonin secretion.

Time of Collection	N =	Mean	Median	Sd	Min	Max	Percentile		
							25th	50th	75th
4:00–6:00	7	141.3	16.5	213	5.7	583.0	11.6	16.5	180.1
6:00–8:00	11	130.8	17.1	246	2.3	831.0	7.4	17.1	134.6
8:00–10:00	13	139.1	60.3	173	4.5	583.0	33.6	60.3	176.9
10:00–12:00	13	119.8	45.1	215	8.9	805.0	18.7	45.1	80.3
12:00–14:00	11	120.5	69.1	117	14.5	343.0	33.4	69.1	164.9
14:00–16:00	13	93.6	47.9	120	4.8	418.0	22.7	47.9	112.2
16:00–18:00	14	160.9	43.3	223	8.3	683.0	20.4	43.3	268.4
18:00–20:00	13	98.4	33.0	121	2.7	351.0	19.2	33.0	171.8
20:00–22:00	8	102.5	53.3	174	1.9	530.0	23.2	53.3	63.0

Sd: standard deviation. Max: maximal value. Min: minimal value. N: number of data points for each time slot.

**Table A2:** Cortisol secretion.

Time of Collection	N =	Mean	Median	Sd	Min	Max	Percentile		
							25th	50th	75th
4:00–6:00	6	7.00	5.59	5.43	1.57	17.20	4.38	5.59	7.48
6:00–8:00	12	5.75	5.14	1.62	3.84	9.44	4.82	5.14	6.25
8:00–10:00	16	5.75	3.48	4.83	1.29	17.58	2.44	3.49	7.79
10:00–12:00	16	2.41	1.93	1.09	1.06	4.63	1.60	1.93	3.06
12:00–14:00	13	3.19	1.99	2.41	0.89	7.81	1.39	1.99	4.87
14:00–16:00	15	2.58	2.18	1.27	1.36	5.35	1.54	2.18	3.08
16:00–18:00	17	2.26	1.70	1.51	0.55	5.53	1.05	1.70	3.03
18:00–20:00	17	2.26	1.70	1.51	0.55	5.53	1.05	1.70	3.03
20:00–22:00	15	1.62	0.87	1.77	0.62	7.47	0.65	0.87	1.76

Sd: standard deviation. Max: maximal value. Min: minimal value. N: number of data points for each time slot.
